# Dental Education

**Published:** 1877-04

**Authors:** C. W. Spalding


### Selections

### DENTAL EDUCATION.

If dentistry is ever to grow to the full stature of a real profession, it must be done by fixing a higher standard of attainment, as a condition of recognition and of reception into its ranks. This work can only be accomplished by dentists themselves; and the main question at issue at this time is, how can this result be best brought about. If we wait for the accession of learned men from other professions for its accomplishment, we shall wait a long time. If again, we rely on the profession of medicine to extend a helping hand, the result in the future will be just what it has been in the past. What then shall be done? I answer, cut loose from all "entangling alliances" with medicine, and proceed to establish ourselves as an independent profession.

So long as there is an element among us that persists in attempting to attach itself (and the rest of us along with it) to the skirts of medicine, so long will dentistry hold, respectively to medicine, an inferior position. This result is inevitable, for the reason that only a small proportion of the candidates for admission into our profession, can afford to incur the outlay of time and money requisite to the study and acquirement of two professions. If one must graduate at a medical school to obtain recognition by the medical fraternity, and also graduate at a dental school, that he may be qnalified for the practice of dentistry, very few will ever comply with the requirement.

Medicine demands that every medical specialist shall first become a regular M. D., and afterwards qualify himself for the peculiar duties of his specialty. Of this we have no right to complain; for dentistry, if placed in the same position relatively to medicine, would demand the same thing. There is no injustice, no hardship, nor want of liberality, on the part of medical men, in insisting on such an educational course, as a condition of admission into their ranks, or as a condition of recognizing any calling as a specialty of medicine. Were they to do less, they would only degrade their own standard of excellence, and bring their own profession into disrepute.

The question then is narrowed down to this: Can such requirements be enforced upon candidates for admission into the ranks of dentistry? If dentistry is to take position as a specialty of medicine, these requirements must be enforced, and the question again recurs, will dental students conform to such requirements? Can a majority of them afford to do so? It may be answered, that if some such restrictions enveloped dental studentship, the number of applicants would be lessened, and that the profession would be the gainer by reason thereof. But it does not always happen that those who can afford the most lavish expenditure of money, promise to make the most useful members. Indeed, many of our most valuable members have been recruited from the non-professional callings of life.

Would it not be better, then, to wholly discard the idea that dentistry is ever to become a specialty of another profession, and to take, forthwith, such action as shall place it in a position of self-reliant independence, friendly towards all, but subservient to none?

Every profession must stand upon its own merits, and can not be helped to a position of public esteem, by the friendly aid of another. In estimating men and things, the community act as an impartial jury, and the verdict of public opinion is almost always just. To deserve well of the public, then, we must qualify ourselves for the performance of our specific duties, in the very best manner practicable, under the circumstances. The only question comes back to us again, can this be done in any other way than by marking out a straightforward but independent course, and pursuing that line of action, to its legitimate consequences? That it would result in good, can scarcely be doubted. That it would end in placing our profession on a firm and independent basis, is equally clear.

Our organization is now entirely separate from that of medicine. We have our local, state and national societies, apart from those of medicine, and their usefulness is in consequence of their separate existence. To be consistent, the advocates of the medical-specialty position, should also propose to merge our societies into those of medicine, and perhaps extend the rule to our periodical literature also.—C. W. SPALDING.—*Missouri Dental Journal.*

